# Large left atrial cavernous hemangioma, a case report

**DOI:** 10.1186/s13019-019-1005-9

**Published:** 2019-11-15

**Authors:** Jian Xu, Hulin Piao, Duo Wang, Yong Wang, Bo Li, Tiance Wang, Zhicheng Zhu, Dan Li, Rihao Xu, Kexiang Liu

**Affiliations:** Department of Cardiovascular Surgery, Second Hospital of Bethune, Jilin University, No. 218 Ziqiang Street, Changchun, 130022 China

**Keywords:** Cavernous hemangioma, Cardiac benign tumor

## Abstract

**Background:**

Cardiac cavernous hemangiomas are extremely rare and usually difficult to be diagnosed for being asymptomatic.

**Case presentation:**

An asymptomatic 56-year-old woman was hospitalized due to a heart mass found by chest computed tomography (CT) during her annual physical examination. Coronary computed tomography angiography (CTA) disclosed a tumorous lesion, located in the left atrial roof and extended to the posterior wall of the aortic root and surrounding the left main coronary artery. However, there was no communicating branches between the hemangioma and coronary artery and no coronary artery stenosis. The tumor was excised with low-frequency electrocautery under cardiopulmonary bypass. The histopathological examination indicated the mass a cavernous hemangioma. The patient was discharged with an uneventful recovery.

**Conclusions:**

Here we presented a rare case of successfully excision of a cavernous hemangioma involving the left atrial roof and left coronary artery. We advocate adequate exposure and complete surgical excision with low-frequency electrocautery to avoid remnants and excessive resection.

## Background

Cardiac hemangiomas are extremely rare benign vascular tumors of the heart, with an incidence of less than 0.03% at autopsy [1]. Histologically, cardiac hemangiomas can be classified into three categories: capillary, cavernous, and arteriovenous hemangiomas [2]. Of these, cardiac cavernous hemangiomas are exceptionally rare, and seldom involving the left atrial and coronary artery. Surgical excision should be performed as early as possible after diagnosis [3]. To our knowledge, only 3 references of surgically treated left atrial roof cavernous hemangiomas have been reported [4–6]. We reported a cavernous hemangioma located in the left atrial roof and involving the left coronary artery.

### Case presentation

**Fig. 1:**
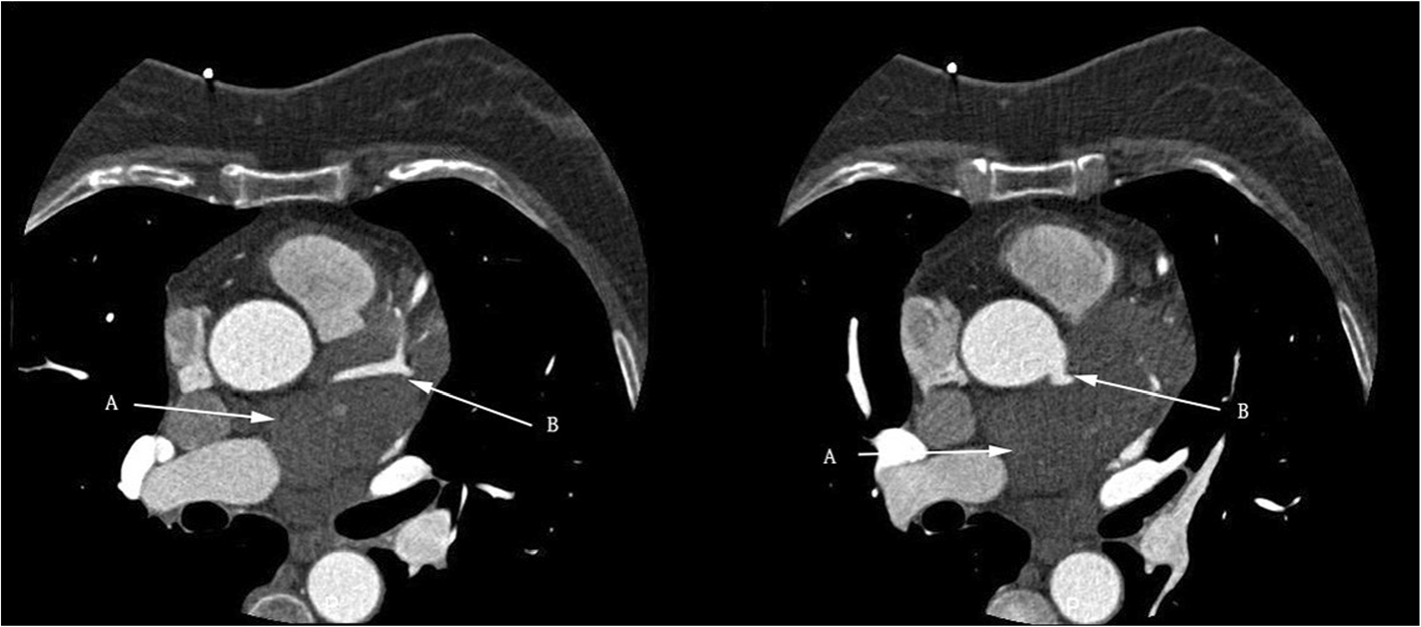
Coronary CTA. A large low-density mass was located in the epicardium of the left atrial roof with ambiguous boundaries. The left coronary artery was not affected (A. Tumor, B. Left coronary artery)

**Fig. 2:**
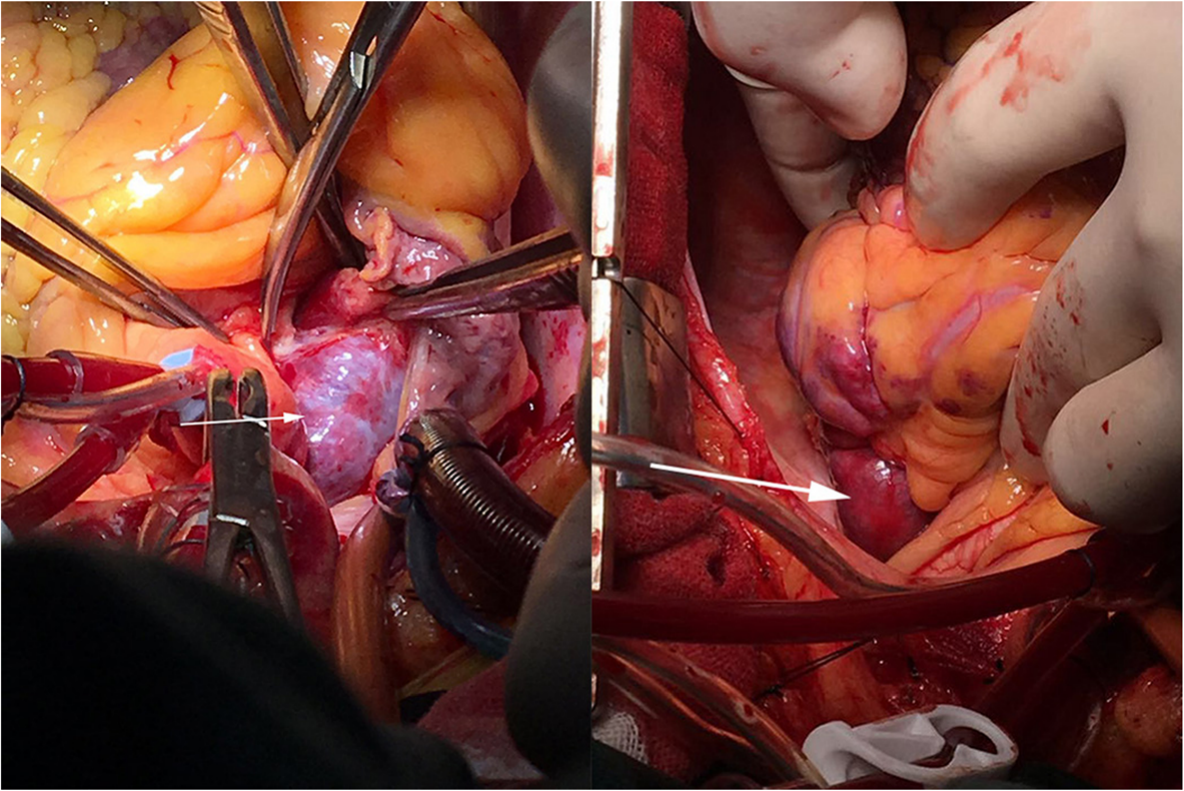
Intraoperative view of the tumor

**Fig. 3:**
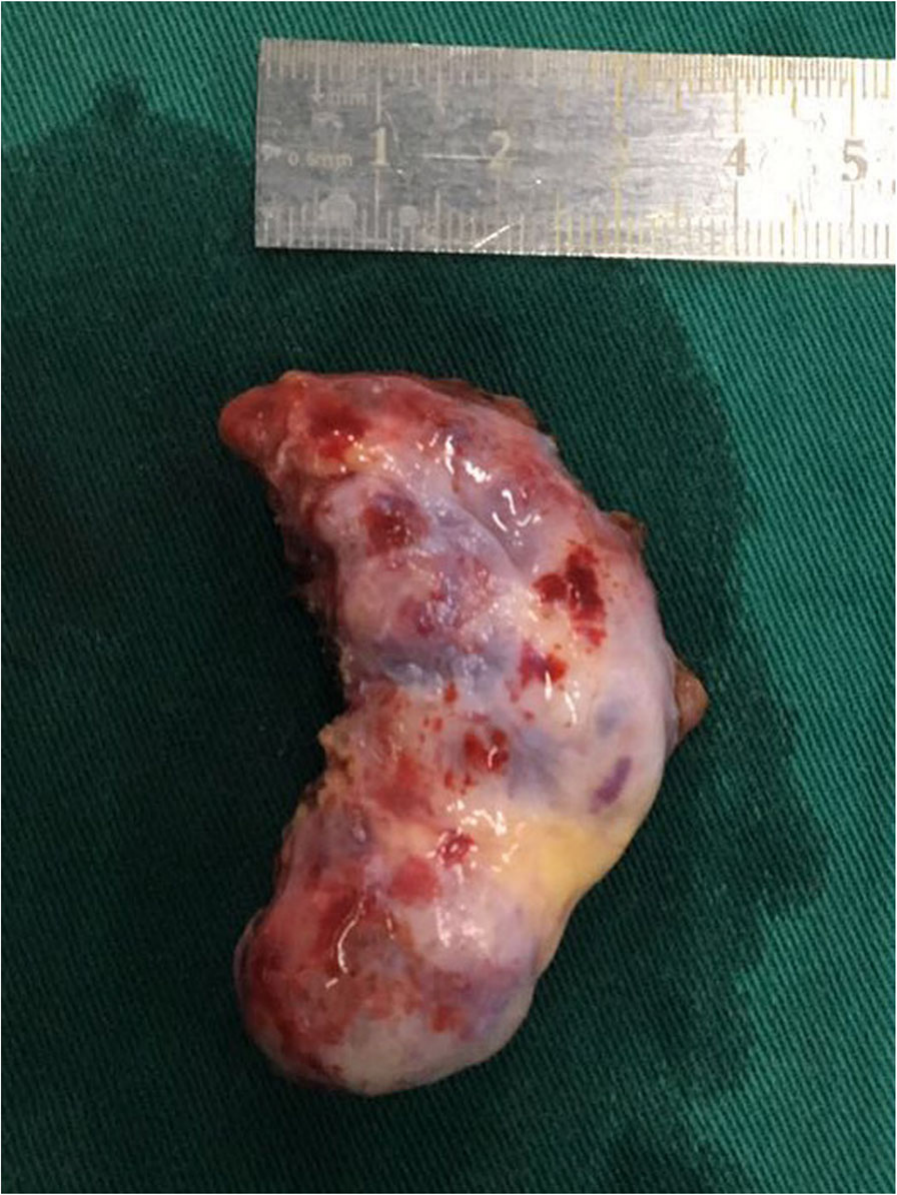
The gross inspection of the tumor. It was a 7 cm × 4 cm × 3 cm, elastic, soft and reddish-brown mass

**Fig. 4:**
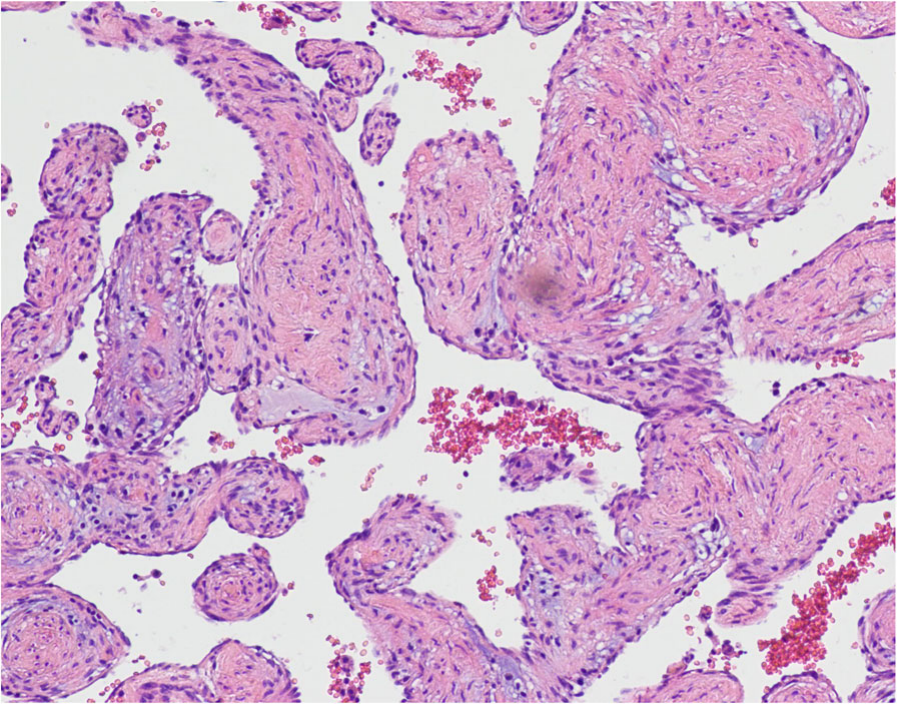
The histopathological examination revealed cavernous hemangioma

**Fig. 5:**
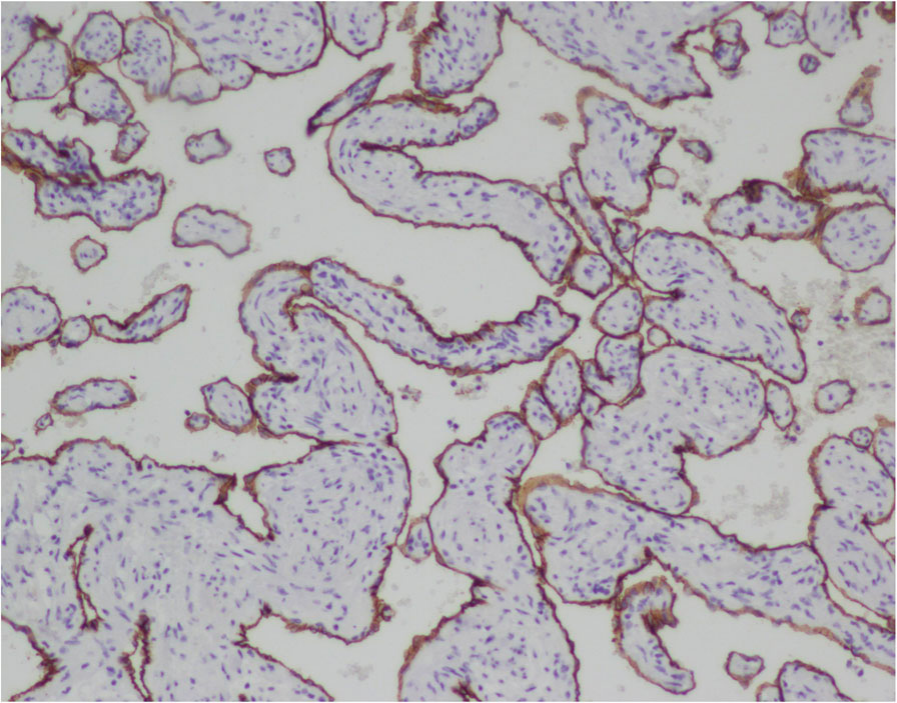
Immunohistochemical staining of the cavernous hemangioma. Cells lining the vessels stained positive with CD31, CD34, supporting their endothelial origin

An asymptomatic 56-year-old female was hospitalized because of a cardiac mass noted by a chest computed tomography (CT) scan during a physical examination. Preoperative transthoracic echocardiography (TTE) revealed an uneven hyperechoic mass, measuring 81 m × 38 mm, located in the left atrium. The mass attached to the interatrial septum adjacent to the mitral annulus but was not hampering blood flow of mitral valve. Preoperative coronary computed tomography angiography (CTA) showed a relatively low-intensity mass (78 mm × 42 mm) in the left atrial roof and extended to the posterior wall of the aortic root. Coronary CTA revealed no communicating branches between the hemangioma and coronary artery or coronary artery stenosis (Fig. 1). Thus, the surgery was performed via a median sternotomy under cardiopulmonary bypass (CPB). After the pericardium was incised, the tumor was found to cover the left atrial roof and extend to the posterior wall of the aortic root (Fig. 2). We successfully excised the tumor with low-frequency electrocautery from the left atrium to the aortic root without injury the coronary artery and the left atrial wall. Left atrium was opened to inspect the invasion status. Its endocardium was smooth and clear of invasion. After heart resuscitation, the patient weaned easily off bypass. On gross inspection, the tumor was a 7 cm × 4 cm × 3 cm, elastic, soft and reddish-brown mass (Fig. 3). Histological examination after hematoxylin-eosin staining demonstrated a tumor comprised multiple dilated vascular channels with endothelial cell lining (Fig. 4). Cells lining in the vessels stained positive with CD31, CD34(Fig. 5), Indicating their endothelial origin. Cytologic examination revealed the presence of numerous mesenchymal and inflammatory cells, and absence of malignant cells.

The patient was discharged 5 days after the operation with an uneventful recovery. Telephone following-up is conducted every 3 months after discharge, the patient claimed no obvious discomfort. TTE 6 months after surgery showed no relapse of the cavernous hemangioma.

## Discussion

Cavernous hemangioma is a rare disease. The clinical presentations of the cavernous hemangiomas of the heart are nonspecific and variable according to the location of the tumor and the resultant cardiac hemodynamic consequences. Manifestations of the symptomatic patients are arrhythmias, heart failure, embolic episodes, pericardial effusion, cardiac tamponade, myocardial ischemia and sudden death [3]. Most patients are asymptomatic, in our case, the patient was diagnosed through imaging during routine physical examination while no complaint was expressed.

Imaging methods are widely applied for diagnosing cardiovascular anomalies and cardiac tumor. Echocardiography is typically used to identify a cardiac mass; but CT and magnetic resonance imaging (MRI) are superior to echocardiography for further characterizing cardiac hemangiomas [7]. Coronary artery angiography can occasionally establish the diagnosis of hemangioma by its characteristic tumor blushing [5]. Definitive diagnosis was made based on postoperative pathological findings. In this case, Preoperative transthoracic echocardiography (TTE) revealed an intracardiac tumor. While the location of the lesion was determined poorly. Coronary CTA was administrated and a tumorous lesion was disclosed and located in left atrial roof. Coronary CTA is an useful method to assess the extent of cardiac hemangiomas and the vascular supply of coronary arteries. It also has a high evaluability and sensitivity in detecting coronary stenosis.

Surgical excision should be performed as early as possible once diagnosed, especially in symptomatic cases [3]. Median sternotomy with cardiopulmonary bypass is universally required. Surgical resection aided by thoracoscopy or mini-thoracotomy can also be taken into consideration according to the mass location and adhesions situation. In this case, the cardiac cavernous hemangioma located in the left atrial roof, extended to the posterior wall of the aortic root and surrounding the left main coronary artery. Median sternotomy was conducted under CPB to provides adequate exposure. The mass extended widely and involved the left coronary artery, which has not been reported in other cases yet. Coronary artery bypass grafting (CABG) and reconstruction of the left atrial wall may need to be conducted in invasive cases [6, 8]. The tumor was carefully excised with low-frequency electrocautery. However, despite of extensive compression and distortion of surrounding structures, the tumor was excised completely without injuring the left coronary artery and left atrial wall.

## Conclusions

In this rare case, our center conducted the excision of a cavernous hemangioma involving the left atrial roof and left coronary artery. We advocate adequate exposure and complete surgical excision with low-frequency electrocautery to avoid remnants and excessive resection.

## Abbreviations

CABG: Coronary artery bypass grafting
CAD: Coronary artery disease
CPB: Cardiopulmonary bypass;
CT: Computed tomography
CTA: Computed tomography angiography
MRI: Magnetic resonance imaging
TTE: Transthoracic echocardiography
